# *Hygrophorus* subsection *Hygrophorus* (Hygrophoraceae, Agaricales) in China

**DOI:** 10.3897/mycokeys.68.53264

**Published:** 2020-06-26

**Authors:** Chao-Qun Wang, Tai-Hui Li, Ming Zhang, Xiao-Lan He, Wei-Qiang Qin, Tie-Zhi Liu, Nian-Kai Zeng, Xiang-Hua Wang, Jian-Wei Liu, Tie-Zheng Wei, Jiang Xu, Yue-Qiu Li, Ya-Heng Shen

**Affiliations:** 1 Guangdong Provincial Key Laboratory of Microbial Culture Collection and Application, State Key Laboratory of Applied Microbiology Southern China, Guangdong Institute of Microbiology, Guangdong Academy of Sciences, Guangzhou 510070, Guangdong, China Guangdong Institute of Microbiology, Guangdong Academy of Sciences Guangdong China; 2 Soil and Fertilizer Institute, Sichuan Academy of Agricultural Sciences, Chengdu 610066, Sichuan, China Soil and Fertilizer Institute, Sichuan Academy of Agricultural Sciences Chengdu China; 3 Jishou University, Zhangjiajie 427000, Hunan, China Jishou University Hunan China; 4 College of Life Sciences, Chifeng University, Chifeng 024000, Inner Mongolia, China Chifeng University Chifeng China; 5 College of Pharmacy-Transgenic Laboratory, Hainan Medical University, Haikou 571199, Hainan, China Hainan Medical University Haikou China; 6 Key Laboratory for Plant Diversity and Biogeography of East Asia, Kunming Institute of Botany, Chinese Academy of Sciences, Kunming 650201, Yunnan, China Kunming Institute of Botany, Chinese Academy of Sciences Kunming China; 7 State Key Laboratory of Mycology, Institute of Microbiology, Chinese Academy of Sciences, Beijing 100101, China Institute of Microbiology, Chinese Academy of Sciences Beijing China

**Keywords:** East Asia, molecular systematics, taxonomy, waxycap, woodwax

## Abstract

Hygrophorus subsect. Hygrophorus has been relatively well-studied in Europe and North America, but studies on the taxa in Asia, particularly in China, are still limited. In this study, phylogenetic overviews of genus *Hygrophorus*, based on the nuclear large subunit (LSU) ribosomal RNA gene and of subsect. Hygrophorus, based on the nuclear ribosomal internal transcribed spacer (ITS) regions were generated. Four new species, i.e. *H. brunneodiscus*, *H. fuscopapillatus*, *H. glutiniceps* and *H. griseodiscus* are described from southern China; and a rarely reported edible species *H. hedrychii* is described in detail, based upon the materials from north-eastern China. The main characteristics of the species under subsect. Hygrophorus worldwide are summarised in a table.

## Introduction

*Hygrophorus* Fr. (Hygrophoraceae, Agaricales, Basidiomycota) is a cosmopolitan fungal genus, mainly distributed in the northern hemisphere. The characteristics that distinguish the genus are: the ectomycorrhizal habit, robust basidiomata, usually viscid pileus surface, waxy, thick and distant lamellae, divergent lamellar trama and white or hyaline thin-walled basidiospores (Candusso 1997, Young 2005). The divergent lamellar trama morphologically distinguishes *Hygrophorus* from the other genera in the family Hygrophoraceae (Singer 1986, Lodge et al. 2014). According to the recent phylogenetic study, *Hygrophorus* can be divided into three subgenera [subg. Camarophylli Fr., subg. Colorati (Bataille) E. Larss. and subg. Hygrophorus], while subg. Hygrophorus is divided into three sections [sect. Discoidei (Bataille) Konrad & Maubl., sect. Hygrophorus and sect. Picearum E. Larss.] and sect. Hygrophorus consists of subsect. Fulventes E. Larss. and subsect. Hygrophorus, which includes the generic type species *H. eburneus* (Bull.: Fr.) Fr. (Lodge et al. 2014).

Morphologically, members in subsect. Hygrophorus share the characteristics of glutinous and white or pallid pileus, almost white and sometimes darkening lamellae, glutinous stipe and *Cossus*-odour, resembling the smell of *Cossus cossus* (Lepidoptera) (Larsson and Jacobsson 2004, Lodge et al. 2014). Five known species are currently included in subsect. Hygrophorus, i.e. *H. cossus* (Sow.) Fr., *H. discoxanthus* (Fr.) Rea, *H. eburneus*, *H. hedrychii* (Velen.) K. Kult. and *H. scabrellus* A. Naseer & A.N. Khalid, according to Larsson and Jacobsson (2004), Lodge et al. (2014) and Naseer et al. (2019).

Phylogenetically, the relationships of subsect. Hygrophorus are still controversial. The ITS-LSU-SSU-RPB2 combined analysis in Lodge et al. (2014) showed that subsect. Hygrophorus was a polyphyletic group; ITS analysis in Endo et al. (2018) showed subsect. Hygrophorus as a polyphyletic group since *H. discoxanthus* was located at the base of sect. Hygrophorus. ITS analysis in Lodge et al. (2014) showed that subsection was mostly monophyletic with an unstable support, but *H. discoideus* should belong to subsect. Discoidei. The ITS-LSU combined analysis in Lodge et al. (2014) and ITS analysis in Naseer et al. (2019) indicated subsect. Hygrophorus as a monophyletic group.

During the authors’ study on the diversity of subsect. Hygrophorus in China, five species were discovered. In order to assess their phylogenetic positions, a phylogenetic overview of genus *Hygrophorus* was conducted, based on all available sequences of the nuclear large subunit (LSU) ribosomal RNA gene from GenBank (Benson et al. 2017) and the newly-obtained Chinese sequences in this study; and a phylogenetic overview of subsect. Hygrophorus was also made with the available sequence data of nuclear ribosomal internal transcribed spacer (ITS) regions from GenBank and UNITE (Nilsson et al. 2019) and the newly-obtained sequences from the Chinese materials. Through morphological comparisons along with the phylogenetic analyses, four new species, i.e. *H. brunneodiscus* from Central South China, *H. fuscopapillatus* and *H. griseodiscus* from south-western China and *H. glutiniceps* from southern China, are firstly introduced in this paper; and the presence of *H. hedrychii* in China is confirmed using molecular data, based on fungal collections from north-eastern China and also described and elucidated, based on macro- and micromorphological observations.

## Materials and methods

### Morphological observation and descriptions

Specimens are deposited in the Mycological Herbarium of Chifeng University (CFSZ), Fungal Herbarium of Hainan Medical University (FHMU), Fungarium of Guangdong Institute of Microbiology (GDGM), Herbarium of Mycology, Academia Sinica (HMAS) and Mycological Herbarium of Soil and Fertilizer Institute, Sichuan Academy of Agricultural Sciences (SAAS). Macro-morphological characteristics are described on the basis of field notes and photos and colour codes followed Kornerup and Wanscher (1978). Micro-morphological characteristics are measured when tissues are mounted in 5% aqueous potassium hydroxide (KOH) and 1% Congo Red solution under an Olympus BX51 microscope (Olympus Co., Tokyo, Japan). Twenty basidiospores and ten basidia from mature lamellae are measured for each specimen, Q refers to the ratio of length to width and Q_m_ refers to the mean value of Q values of all spores; pileipellis and stipitipellis are observed using the hand-sliced tissues approximately at the surface of the mid-radius of the pileus and the middle of the stipe length, respectively. Description terms for morphological characteristics mainly follow Vellinga (1988).

### DNA extraction, PCR amplification and sequencing

The total genomic DNA extracted from dry samples using a HiPure Fungus DNA Mini kit (Megen Biotech Co. Ltd., Guangzhou, China) according to the manufacturer’s instructions. Gene regions of the large subunit (LSU) and the internal transcribed spacer (ITS) nuclear ribosomal RNA gene are amplified by Polymerase Chain Reactions with the primers LR5 and LR0R (https://sites.duke.edu/vilgalyslab/rdna_primers_for_fungi/) and ITS1/ITS5 and ITS4 (White et al. 1990, Gardes and Bruns 1993), respectively. Sequencing of both directions was performed on an ABI 3730 sequencer analyser (Applied Biosystems, Foster City, CA, USA) using the same PCR primers at Beijing Liuhe Co. Ltd. Raw sequences are assembled by using SEQMAN version 7.1.0 of LASERGENE software (DNAStar, Madison, WI, USA). Newly-obtained consensus sequences are deposited in GenBank (Table 1).

**Table 1.** Taxa, vouchers, geographic origin and GenBank accession numbers of the newly-generated sequences in this study.

Taxon	Voucher	Geographic origin	ITS	LSU
*H. brunneodiscus*	GDGM73213	China: Hunan	MN378318	MT093623
	GDGM75489	China: Hunan	MN378317	MT093622
	GDGM76934	China: Hunan	MT093605	MT093621
*H. eburneus*	GDGM70059 (BHS2011-11)	USA	MT093608	
*H. fuscopapillatus*	GDGM44412	China: Sichuan	MN378337	MT093625
	LJW1858	China: Yunnan	MT093606	MT093626
	XHW6609	China: Yunnan	MT093607	MT093627
*H. griseodiscus*	FHMU1578 (Zeng2452)	China: Hainan	MN378311	
	FHMU2013 (Zeng3052)	China: Hainan	MN378312	MT093614
	GDGM42140	China: Guangdong	MN378310	MT093619
	GDGM42188	China: Guangdong	MN378313	MT093618
	GDGM42217	China: Guangdong	MN378309	MT093617
	GDGM45220	China: Hainan	MN378315	MT093620
	GDGM53153	China: Jiangxi	MT093603	MT093615
	GDGM53496	China: Hunan	MN378314	
	HMAS273294	China: Guangdong	MT093604	
	SAAS462	China: Sichuan	MN378338	MT093624
*H. hedrychii*	CFSZ2559	China: Inner Mongolia	MT093610	
	CFSZ2851	China: Inner Mongolia	MN378306	MT093628
	CFSZ4268	China: Inner Mongolia	MT093609	
	CFSZ10761	China: Inner Mongolia	MT093611	
	CFSZ18159	China: Inner Mongolia	MN378308	MT093629
	CFSZ18269	China: Inner Mongolia	MN378307	MT093630

### Sequence alignments and phylogenetic analyses

For the LSU dataset, the newly-obtained sequences and all available *Hygrophorus* sequences longer than 300 bps from GenBank are treated as ingroups and the sequences of *Cantharocybe gruberi* (A.H. Sm.) H.E. Bigelow & A.H. Sm. are selected as outgroup, based on Razaq et al. (2014) and Naseer et al. (2019); for the ITS dataset, the newly-obtained sequences and the downloaded sequences from GenBank and UNITE longer than 300 bps and of subsect. Hygrophorus are combined as ingroups and the sequences of *H. arbustivus* Fr. are selected as outgroups, since *H. arbustivus* is the type species of the sister group subsect. Fulventes, according to Lodge et al. (2014). Both datasets are combined, using GENEIOUS software (Biomatters Ltd.) and aligned by MAFFT (Multiple Alignment using Fast Fourier Transform) online service version 7 (Katoh et al. 2017). Maximum Likelihood analyses are performed by IQ-TREE web server with 1000 rapid bootstrap (BS) replicates (Trifinopoulos et al. 2016). The trees are viewed and edited in ITOL (Interactive Tree of Life) web server (Letunic and Bork 2016).

## Results

### Molecular phylogenetic results

**Figure 1.**
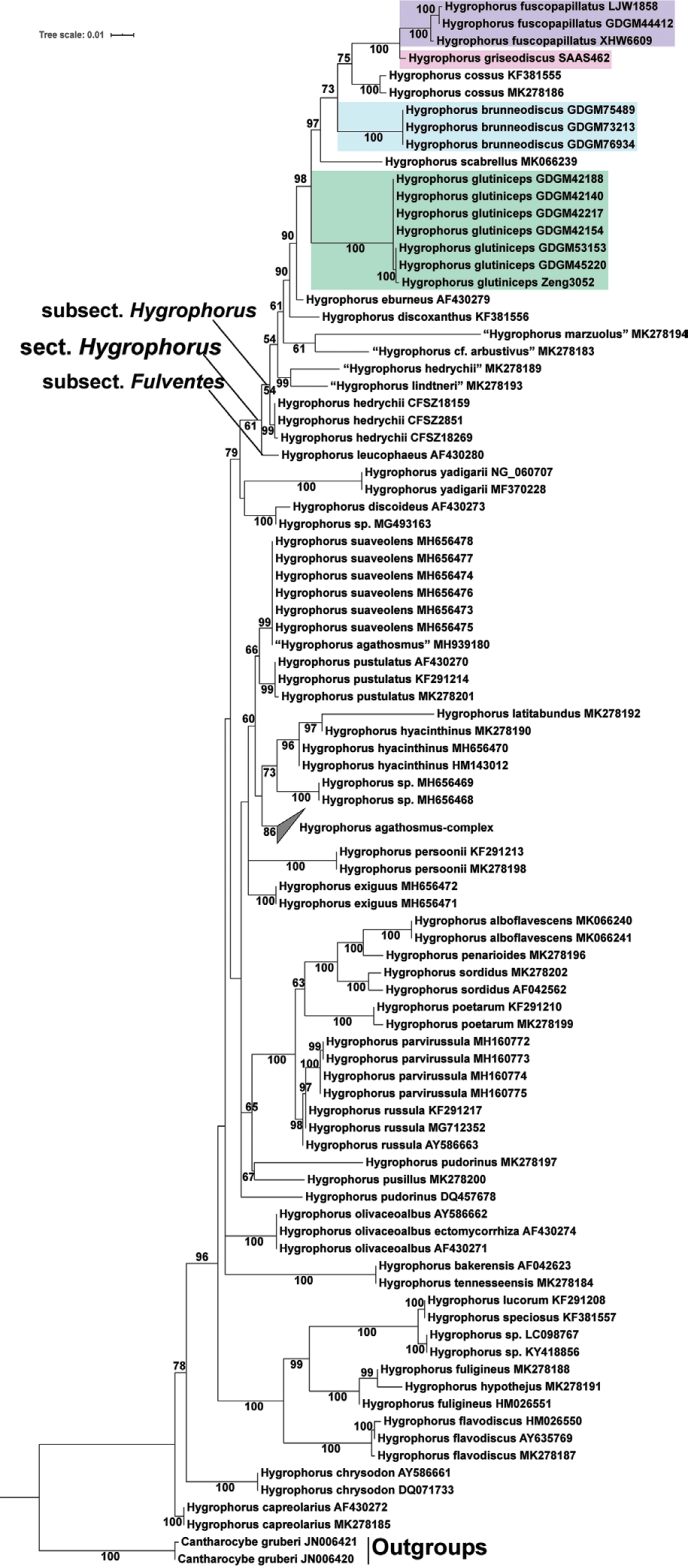
Phylogram showing the interspecific relationships under the genus *Hygrophorus* inferred from a LSU dataset using IQ-tree. Sequences of *Cantharocybe gruberi* were selected as outgroups. Bootstrap values greater than 50% are indicated around the branches. For downloaded sequences, specimen names and GenBank accession numbers are presented; for newly-generated sequences, species names and specimen vouchers are listed. Four newly-described species’ sequences are highlighted in colour; sequences with quotation marks are incorrect names.

For the aligned LSU dataset, 121 sequences with 980 sites were included, amongst them 119 *Hygrophorus* sequences are placed in the ingroups and two sequences of *Cantharocybe gruberi* are in the outgroups. In the LSU phylogenetic tree (Fig. 1), both sect. Hygrophorus and subsect. Hygrophorus are presented as monophyletic groups with low support values, 61% BS and 54% BS, respectively; in addition, the sequences named as “*H. lindtneri*” (MK278193), “*H. marzuolus*” (MK278194) and “H. cf. arbustivus” (MK278183) are present within these clades. Three sequences from three samples (GDGM44412, LJW1858 and XHW6609) of *H. fuscopapillatus* are present as a monophyletic clade with high support value (100% BS), *H. griseodiscus* is present as a sister clade to *H. fuscopapillatus* with 100% BS, and, together, they form a sister clade to *H. cossus* with 75% BS; three sequences of *H. brunneodiscus* samples (GDGM73213, GDGM75489 and GDGM76934) and seven sequences of *H. glutiniceps* samples (GDGM42140, GDGM42154, GDGM42188, GDGM42217, GDGM45220, GDGM53153 and Zeng3052) form two independent monophyletic clades with high support values (100% BS) respectively; in addition, three sequences of *H. hedrychii* specimens (CFSZ2851, CFSZ18159 and CFSZ18269) form a monophyletic clade with high support value (99% BS).

**Figure 2.**
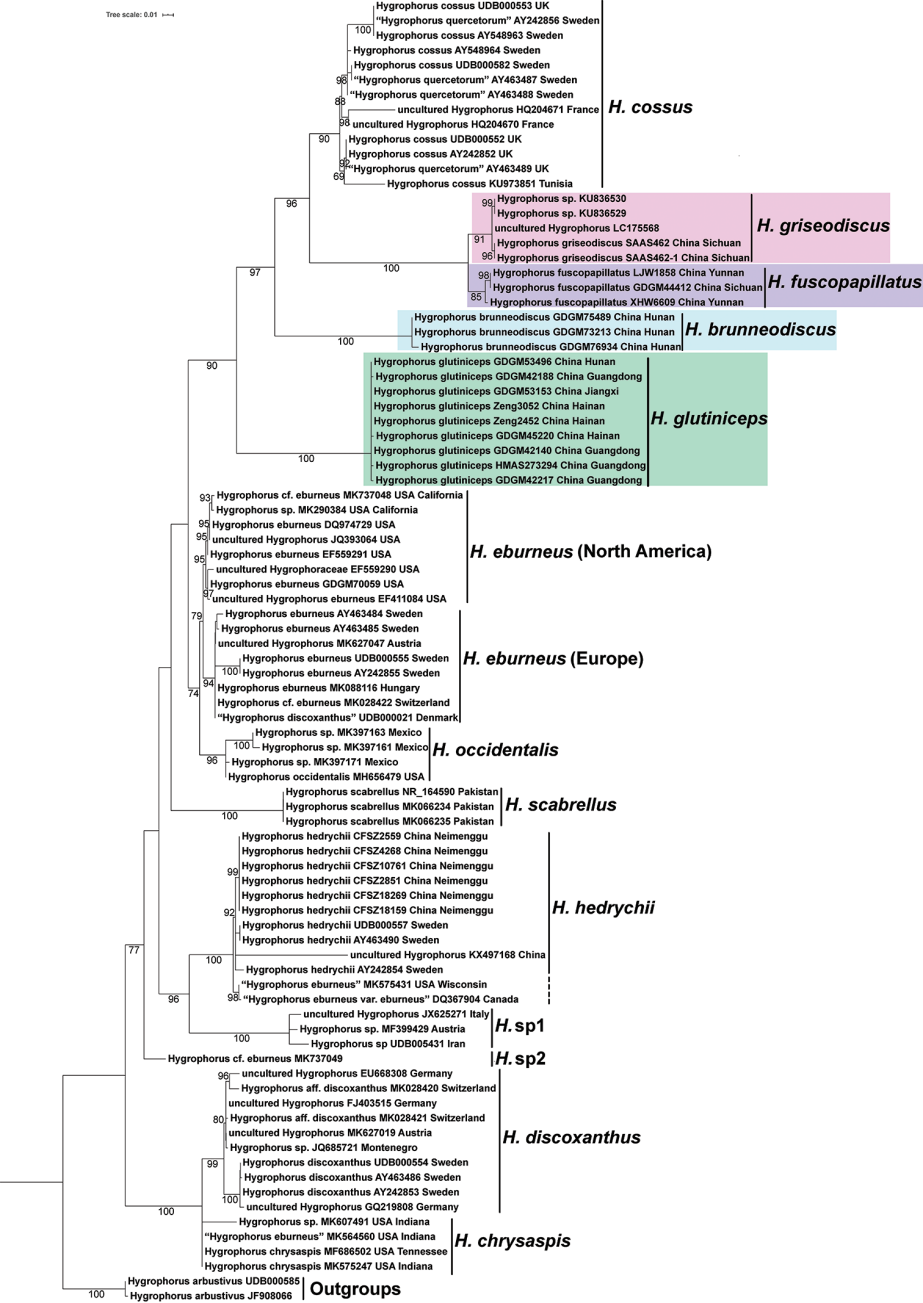
Phylogram of species under subsect. Hygrophorus inferred from an ITS dataset using IQ-tree. Sequences of *Hygrophorus arbustivus* were selected as outgroups. Bootstrap values greater than 50% are indicated around the branches. For downloaded sequences, specimen names, GenBank accession numbers and locations are presented; for newly-generated sequences, species names, specimen vouchers and locations are listed. Four newly-described species’ sequences are highlighted in colour; sequences with incorrect names are marked with quotation marks.

For the aligned ITS dataset, 88 sequences with 689 sites were included, two sequences of *H. arbustivus* are treated as the outgroups and 86 sequences of subsect. Hygrophorus are within the ingroups. At least thirteen respectively-supported clades that correspond to different species were recovered in the ITS phylogenetic tree (Fig. 2), five of them coming from China. Two newly-generated sequences of *H. griseodiscus* from two different fruit-bodies of SAAS462 fall together in a supported clade with two GenBank downloaded sequences (KU836529–30) from Sichuan province in China and an uncultured *Hygrophorus* sequence (LC175568) from Hokkaido in Japan and the support value of the *H. griseodiscus*-clade is 91% BS. Three sequences from samples (GDGM44412, LJW1858 and XHW6609) of *H. fuscopapillatus* form a clade with 85% BS. Three sequences of *H. brunneodiscus* samples (GDGM73213, GDGM75489 and GDGM76934) form a clade with strong support value (100% BS). Nine sequences of *H. glutiniceps* specimens (GDGM42140, GDGM42188, GDGM42217, GDGM45220, GDGM53153, GDGM53496, HMAS273294, Zeng2452 and Zeng3052) are also clustered with strong support value (100% BS). Six newly-generated sequences of *H. hedrychii* samples (CFSZ2559, CFSZ2851, CFSZ4268, CFSZ10761, CFSZ18159 and CFSZ18269) from Inner Mongolia Autonomous Region of China are clustered with other *H. hedrychii* sequences from China (KX497168) and Sweden (AY242854 and AY463490) with 92% BS; in addition, MK575431 named as “*H. eburneus*” from Wisconsin in USA and DQ367904, labelled as “H. eburneus var. eburneus” from Canada, form a clade with 98% BS, this North American clade being the sister clade with the Eurasian *H. hedrychii*-clade with 100% BS.

### Species of subsect. Hygrophorus known from China

According to the molecular phylogenetic analyses (Figs 1, 2) and morphological examinations on the Chinese specimens in this study, at least five species of Hygrophorus subsect. Hygrophorus are present in China, including *H. brunneodiscus*, *H. fuscopapillatus*, *H. glutiniceps*, *H. griseodiscus* and *H. hedrychii*. Although *H. cossus* and *H. eburneus* of subsect. Hygrophorus have been reported in China (Chen and Li 2013), but they are not described in the present study, because no DNA sequences or fresh specimens of these two species have been obtained from China in this study.

### Taxonomy

#### Hygrophorus brunneodiscus

Taxon classificationFungiAgaricalesHygrophoraceae

C.Q. Wang & T.H. Li sp. nov.

BF3EE9CE-E372-5912-8ACC-2DBB84DEC9A2

Figure 3

##### Typification.

China, Hunan Province, Zhangjiajie City, Zhangjiajie Campus of Jishou University, on the ground of a forest dominated by *Quercus fabri* and *Q. serrata*, elev. ca. 220 m, 29°8'24"N, 110°27'42"E, 26 May 2019, W.Q. Qin (GDGM73213, Holotype!), ITSMN378318.

##### Etymology.

“*brunneo*-”: brown, “-*discus*”: pileus disc. The species epithet “*brunneodiscus*” (Lat.) refers to the brown pileus disc of this new taxon.

##### Diagnosis.

*Hygrophorus brunneodiscus* differs from *H. cossus* by the smaller pileus (20–50 mm broad), brownish pileus disc, thinner stipe (4–7 mm wide) and the shorter basidia (32–48 µm long); differs from *H. fuscopapillatus* and *H. griseodiscus* by the low elevation distribution and brown pileus disc.

**Figure 3.**
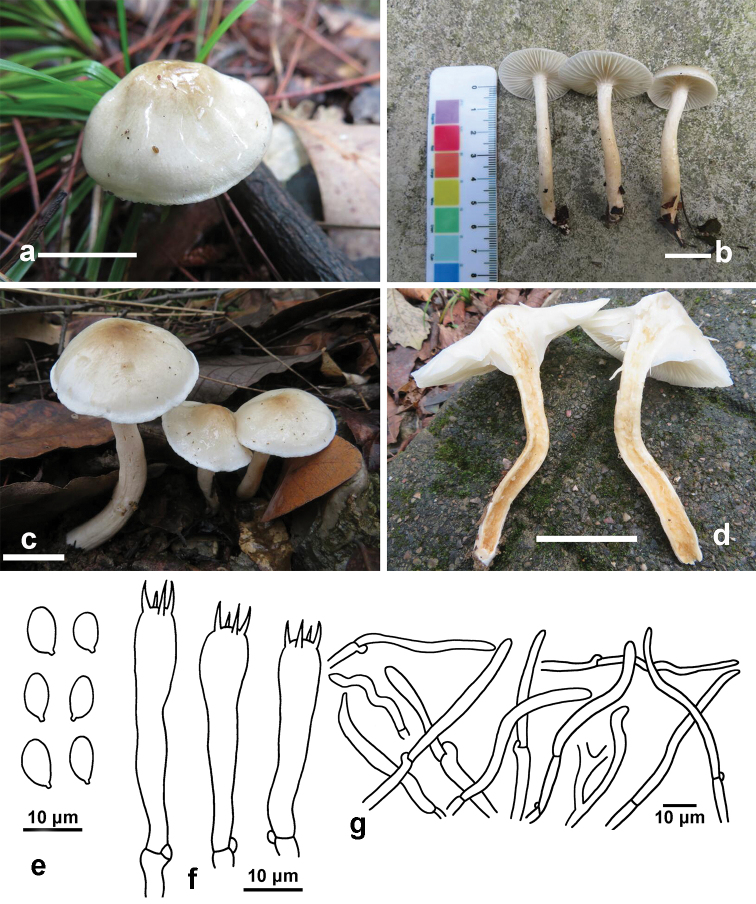
*Hygrophorus brunneodiscus*. **a, b** Basidiomata (GDGM73213, Holotype) **c, d** Basidiomata (GDGM75489) **e** Basidiospores (GDGM73213) **f** Basidia (GDGM73213) **g** Elements of pileipellis (GDGM73213). Scale bars: 2 cm (**a–d**).

##### Description.

*Pileus* 20–50 mm broad, hemispherical or conical with a slightly involute or slightly revolute margin when young, becoming convex with an expanded margin when mature, whitish to brownish as a whole, brownish-orange, light brown, yellowish-brown (5C4–5, 5D4–5, 5E5–8) at the disc (about one fourth part of the radius from the centre to the margin), becoming paler to greyish-yellow (4B3–4), greyish-white (4B1) or whitish outwards and white at the margin or with a white marginal zone of 1–2 mm wide, viscid, covered with a glutinous layer of transparent materials when wet. *Lamellae* short decurrent to decurrent, white, waxy, with 36–40 complete lamellae and 1–3 lamellulae between two complete lamellae; lamella edge concolorous, entire. *Stipe* 40–90 × 4–7 mm, cylindrical, hollow, nearly equal to slightly thinner at apex and tapering towards the base; pale yellow to greyish-yellow (4A3, 4B3), white to yellowish-white (4A2) at apex, sometimes white at base; sticky, covered with a layer of transparent materials when wet, easily-sticking debris on the slime layer, usually fibrillose or with scattered white fibrillose dots at apex. *Context* thin, white to light brown, with slight fishy odour.

*Basidiospores* (6.5)7–9(9.5) × 4–5.5(6) μm [mean length = 7.6 µm, mean width = 4.6 µm], Q = (1.3)1.4–2(2.1), Qm = 1.68, ellipsoid to oblong, smooth. *Basidia* 32–48 × 6–8.5 µm, Q = 4.1–6.6, Qm = 5.3, clavate, thin-walled, 4-spored, with sterigmata 5.5–7(9) µm long. *Pileipellis* an ixotrichoderm, composed of septate cylindrical hyphae, covered with a gelatinous layer; hyphae thin-walled, 2.5–5 μm wide, slightly yellowish and glutinous in KOH. *Hymenophoral trama* divergent, composed of septate, thin-walled and cylindrical hyphae; hyphal cells 45–70 × 6–10 μm, hyaline. *Clamp connections* present.

##### Habit, habitat and distribution.

Solitary to scattered, on the ground of subtropical broad-leaf forest dominated by *Quercus*, so far only known from Hunan Province in South Central China.

##### Additional specimens examined.

China, Hunan Province, Zhangjiajie City, Zhangjiajie Campus of Jishou University, 26 October 2018, W.Q. Qin (GDGM75489); ibidem, 30 June 2019, W.Q. Qin (GDGM76934).

##### Remarks.

*Hygrophorus brunneodiscus* is characterised by its brown tint on pileus disc, sticky pileus and stipe surface, basidiospores (6.5)7–9(9.5) × 4–5.5(6) μm, basidia 32–48 × 6–8.5 µm and subtropical and low elevation distribution.

*Hygrophorus brunneodiscus* can be easily recognised within subsect. Hygrophorus for its brownish colour on the pileus disc. Apart from that, *H. cossus* differs from *H. brunneodiscus* by having pale ochraceous grey colour at the pileus centre and more slender basidia (48–60 × 7–8.5 μm) (Larsson and Jacobsson 2004). *Hygrophorus discoxanthus* differs in the pure white mature basidiomata as young, rusty-brown pileus margin and discolouration of rusty brown at the lamellar edge and different host-association (with *Fagus*) (Candusso 1997, Larsson and Jacobsson 2004). *Hygrophorus eburneus* differs from it by having more carnose pileus and thicker stipe (4–10 mm wide), forming an ectomycorrhizal relationship with *Fagus* (Larsson and Jacobsson 2004). *Hygrophorus hedrychii* differs from it by having larger and more robust fruit-bodies (pileus 30–80 mm in diam. and stipe 5–10 mm in width), forming an ectomycorrhizal relationship with *Betula* (Larsson and Jacobsson 2004, Campo 2015). *Hygrophorus scabrellus* differs from it by owning smaller (24–28 mm in diam.) and off-white with dark green colour pileus, smaller basidiospores (mean length = 6.5 µm, mean width = 3.84 µm) and distribution in temperate forests under *Quercus* trees (Naseer et al. 2019).

#### Hygrophorus fuscopapillatus

Taxon classificationFungiAgaricalesHygrophoraceae

C.Q. Wang & T.H. Li sp. nov.

1E2DEEE9-2C05-59CB-AECB-474DCF4D6EC4

Figure 4

##### Typification.

China, Sichuan Province, Panzhihua City, Yanbian County, Gesala Ecotourism Area, elev. ca. 2900 m, 27°08'10"N, 101°11'33"E, 25 August 2013, M. Zhang & C.Q. Wang (GDGM44412, Holotype!), ITSMN378337.

##### Etymology.

“*fusco*-”: dark brown, “-*papillatus*”: papillate. The species epithet “*fuscopalillatus*” (Lat.) refers to the dark brown or grey brown papilla on the pileus of the new species.

##### Diagnosis.

Differs from *H. griseodiscus* by the host association with Fagaceae, solitary habit, adnate to subdecurrent lamellae and slightly smaller basidispores measuring (6)7–9.5(10) × (4)4.5–5.5(6) µm. The ITS sequence is 95% similar to *H. griseodiscus*.

**Figure 4.**
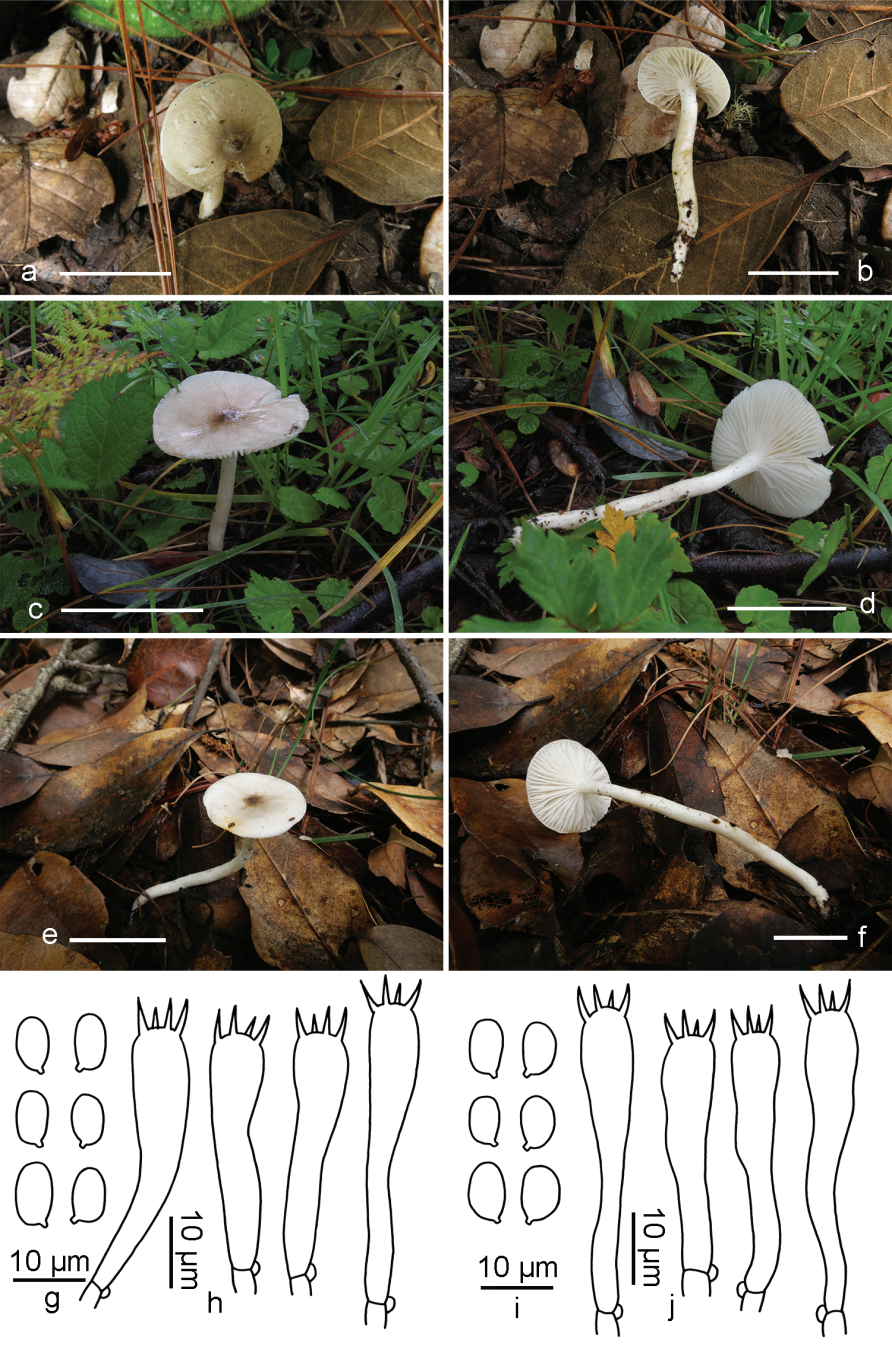
*Hygrophorus fuscopapillatus*. **a, b** Basidiomata (GDGM44412, Holotype) **c, d** Basidiospores Basidiomata (LJW1858) **e, f** Basidiospores and Basidia (XHW6609) **g, h** Basidiospores and Basidia (GDGM44412) **i, j** Basidiospores and Basidia (LJW1858). Scale bars: 2 cm (**a–f**).

##### Description.

*Pileus* 20–30 mm broad, convex to hemispherical when young, applanate to plano-concave when mature, with a papilla or small umbo in the centre, white to pale grey (1B1), grey or brownish-grey to olive brown (4E2–3,4F1–3) at papilla, gradually becoming lighter from centre to margin, white to pale grey (1B1) at margin, glutinous when wet; margin even, occasionally split. *Lamellae* adnate to subdecurrent, white, thick, with 30–36 complete lamellae per pileus and 1–3 lamellulae between two entire lamellae. *Stipe* 40–60 × 4–5 mm, cylindrical, white to yellowish-grey (4B2), covered by a glutinous layer. *Context* thin, whitish.

*Basidiospores* (6)7–9.5(10) × (4)4.5–5.5(6) µm [mean length = 8.2 µm, mean width = 5 µm], Q =1.3–1.9, Qm =1.65, broadly ellipsoid, ellipsoid to oblong, smooth, hyaline. *Basidia* (32)35–46(48) × (6)6.5–8.5(9) µm, Q = 4.4–6.8, Qm = 5.5, clavate, thin-walled, 4-spored, with sterigmata 5–7.5 µm long. *Pileipellis* an ixotrichoderm, composed of septate and thin-walled cylindrical hyphae, covered with a gelatinous layer; hyphal cells 3–5 μm broad. *Hymenophoral trama* divergent, composed of septate, thin-walled and cylindrical hyphae; hyphal elements 13–31.5 μm broad. *Clamp connections* present.

##### Habit, habitat and distribution.

Solitary, on the ground of Fagaceae-dominated forests, so far only known from Sichuan and Yunnan provinces in Southwest China.

##### Additional specimens examined.

China, Yunnan Province, Yulong County, Jade Dragon Snow Mountain, Lijiang Alpine Botanic Garden, on the ground of *Quercus pannosa* dominated forest, elev. ca. 3267 m, 27°00'02"N, 100°10'52"E, 31 August 2019, J.W. Liu (LJW1858); Binchuan County, Jizushan, on the ground of *Castanopsis* and *Lithocarpus* dominated forest, elev. ca. 2853 m, 25°58'06"N, 100°21'39"E, 18 September 2019, X.H. Wang (XHW6609).

##### Remarks.

*Hygrophorus fuscopapillatus* is distinguished by the solitary basidiomes, the brownish-grey to olive brown papilla in the pileus centre, the broadly ellipsoid, ellipsoid to oblong basidiospores measuring (6)7–9.5(10) × (4)4.5–5.5(6) µm.

Amongst the members of subsect. Hygrophours, *H. griseodiscus* closely resembles *H. fuscopapillatus*; however, *H. griseodiscus* differs from *H. fuscopapillatus* by the host association with Pinaceae, the larger pileus (2–4.5 cm broad), the emarginate lamellae with decurrent tooth, the larger basidispores measuring (7)8–10(10.5) × (4)4.5–6(6.5) µm and the broader basidia (7–11 mm broad). In addition, *H. brunneodiscus* is distinguished from *H. fuscopapillatus* by the broader pileus (2–5 cm in diam.) and the brownish pileus disc. *Hygrophorus cossus* is separated by the larger pileus (3–9 cm in diam.), greyish-white lamellae with a cream yellow tint and a thicker stipe (0.6–2 cm broad) (Larsson and Jacobsson 2004). *Hygrophorus discoxanthus* differs by the rusty brown lamellae when mature and the wider stipe (up to 1.2 cm broad); *H. eburneus* is distinguished by the gregarious habit, the more robust basidiomes and the white pileus (Candusso 1997, Larsson and Jacobsson 2004). *Hygrophorus glutiniceps* differs by the subtropical to tropical distribution and smaller basidiospores measuring (5)6–8.5(10) × (3.5)4–6 µm. *Hygrophorus hedrychii* is separated by the larger basidiomes (pileus up to 8 cm in diam.) and the reddish-yellow pileus centre (Larsson and Jacobsson 2004). *Hygrophorus scabrellus* is distinguished by the off-white with dark green pileus, the off-white to beige lamellae and the much smaller basidiospores measuring 6.5 × 3.84 µm (Naseer et al. 2019).

#### Hygrophorus glutiniceps

Taxon classificationFungiAgaricalesHygrophoraceae

C.Q. Wang & T.H. Li sp. nov.

45DB6EAA-085A-56E2-AAFC-AFBFA2A15E8B

Figures 5 , 6

##### Typification.

China, Guangdong Province, Guangzhou City, Tianluhu Forest Park, on the ground of a forest dominated by *Castanopsis fissa*, elev. ca. 250 m, 23°13'39"N, 113°25'53"E, 6 September 2012, M. Zhang (GDGM42188, Holotype!), ITSMN378313.

##### Etymology.

“*glutini*-”: glutinous, “-*ceps*”: pileus. The species epithet “*glutiniceps*” (Lat.) refers to the glutinous surface of pileus.

##### Diagnosis.

*Hygrophorus glutiniceps* differs from *H. discoxanthus* by the subtropical to tropical distribution, the darker lamellae and the thinner stipe (3–6 mm broad).

**Figure 5.**
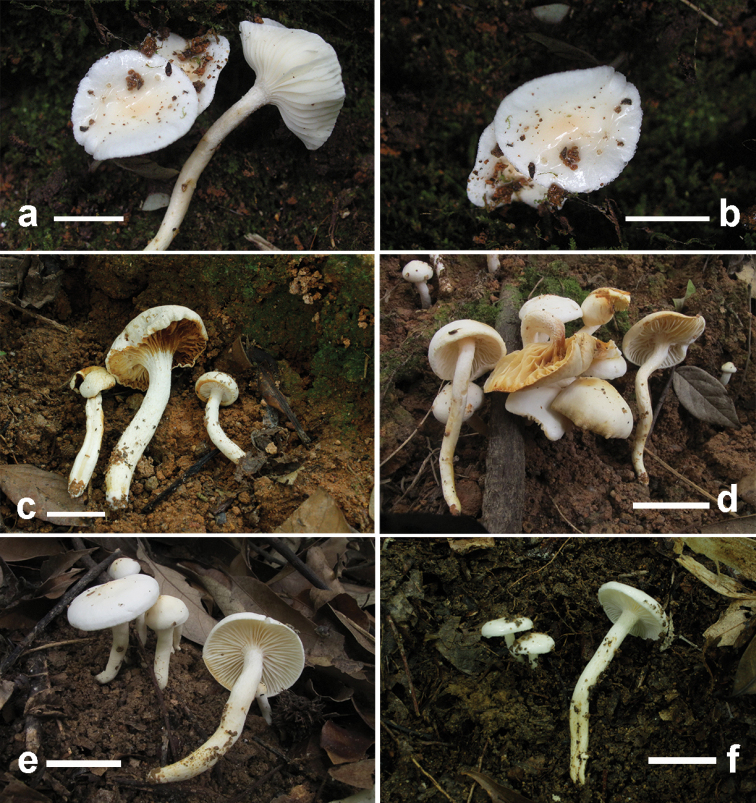
Basidiomata of *Hygrophorus glutiniceps*. **a, b** GDGM42140 **c** GDGM42154 **d** GDGM42188 (Holotype) **e** GDGM42217 **f** GDGM45220. Scale bars: 2 cm.

##### Description.

*Pileus* 8–40 mm broad, hemispherical to convex, often with an inconspicuous umbo and a usually involute margin when young, broad-convex to depressed with a slightly incurved to rarely uplifting margin when mature, covered with a layer of gelatine-like or transparent gluey materials when wet, white with cream or light yellow to orange tint (4A2–4) at disc, becoming light to yellowish-brown (5E8) with age (especially at margin and in wounded area), usually brownish-orange (5C5) to brown (6E8) when exsiccated. *Lamellae* broadly adnate or with decurrent tooth when young, short to moderately decurrent when mature, white when young, then changing to ochraceous or even brown (6E8), waxy when wet, with 25–30 complete lamellae reaching to stipe and 1–3 lamellulae between two entire lamellae. *Stipe* 25–60 × 3–6 mm, equal but often thinner at the base, cylindrical but usually curved, white, occasionally with yellowish tint (2A2), covered with transparent glutinous materials when wet. *Context* thin, white when young, changing to brown when old.

*Basidiospores* (5)6–8.5(10) × (3.5)4–5.5(6) µm [mean length = 7.1 µm, mean width = 4.7 µm], Q = (1.2)1.3–1.77(2), Q_m_ = 1.52, ellipsoid to oblong, smooth, hyaline. *Basidia* 35–47 × 5–8.5 µm, Q values usually more than 5, clavate, thin-walled, 4-spored, with sterigmata up to 7.5 µm long, hyaline. *Pileipellis* an ixotrichoderm, composed of septate hyphae, usually covered with a gelatinous layer; hyphae thin-walled, 3–5 μm wide, with yellowish gluten in KOH. *Stipitipellis* an ixotrichoderm, hyphae 3–5 μm wide, similar to those of pileipellis. *Hymenophoral trama* divergent, composed of septate, thin-walled and cylindrical hyaline in 4–17 μm diam. *Clamp connections* present.

##### Habit, habitat and distribution.

Gregarious to scattered, on the ground of subtropical broad-leaf forest dominated by *Castanopsis*, currently only known from subtropical to tropical areas of China.

##### Additional specimens examined.

China, Guangdong Province, Guangzhou City, Tianluhu Forest Park, 6 September 2012, M. Zhang (GDGM42217); ibidem, 6 September 2012, J. Xu (GDGM42140). Hainan Province, Changjiang County, 3 July 2013, M. Zhang (GDGM45220); Baisha County, Yinggeling National Nature Reserve, elev. ca. 600 m, 1 August 2015, N.K. Zeng 2452 (FHMU1578); Ledong County, Yinggeling National Nature Reserve, elev. ca. 650 m, 4 June 2017, N.K. Zeng 3052 (FHMU2013).

##### Remarks.

*Hygrophorus glutiniceps* is macro-morphologically characterised by its subtropical-tropical distribution, white and sticky pileus and stipe, darkening lamellae when mature or wounded. The size of basidiospores [(5)6–8.5(10) × (3.5)4–5.5(6) µm] and basidia [35–47 × 5–8.5 µm] can be used to confirm the recognition. The association with *Castanopsis fissa* is also helpful for its identification.

*Hygrophorus glutiniceps* can be morphologically distinguished from closely-related species by the following differences. *Hygrophorus cossus* looks different from *H. glutiniceps* in the different ectomycorrhizal connection (with *Quercus*), the temperate distribution, the longer basidiospores [(7–9.5 μm long in Candusso (1997), 7–9 μm long in Larsson and Jacobsson (2004)] and the higher ratio of length to width [Qm = 1.7–1.75 in Candusso (1997)]. *Hygrophorus eburneus* differs in the different host-connection (with *Fagus*), thicker stipe (7–10 mm in width) and larger basidiospores (8–10 × 4.5–5.5 μm) with larger Qm (1.78–1.82); *H. discoxanthus* differs in the different host-connection (with *Fagus*), the more rusty brown tint on pileus and lamellae and the thicker stipe (5–12 mm in width); *H. hedrychii* differs in the different host-connection (with *Betula*) and larger pileus (30–60 mm broad); *H. laurae* Morgan has a much larger basidioma (with pileus 20–40 mm broad) and a wash of red or brown on the disc; and H. laurae var. decipiens Peck, described from New York, USA, differs in the absence of the pileus discolouration when dry and the nearly unchangeable lamellae (Morgan 1883, Peck 1905, Candusso 1997, Larsson and Jacobsson 2004).

**Figure 6.**
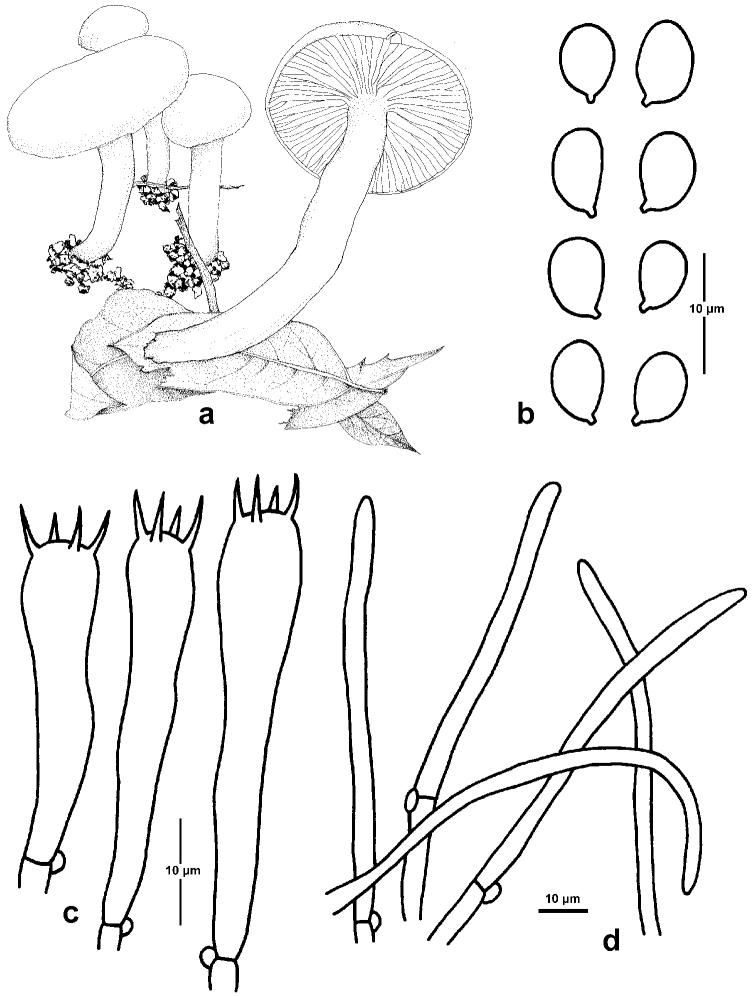
Macro- and microscopic features of *Hygrophorus glutiniceps*. **a** Basidiomata (GDGM42217) **b** Basidiospores (GDGM42188) **c** Basidia (GDGM42188) **d** Terminal cells of pileipellis (GDGM42188).

#### Hygrophorus griseodiscus

Taxon classificationFungiAgaricalesHygrophoraceae

C.Q. Wang & T.H. Li sp. nov.

CAC56DA1-0811-5D11-B489-48009F008348

Figure 7

##### Typification.

China, Sichuan Province, Jiuzhaigou, elev. ca. 3100 m, 11 September 2012, X.L. He (SAAS462, Holotype!), ITSMN378338.

##### Etymology.

“*griseo*-”: grey, “-*discus*”: pileus. The species epithet “*griseodiscus*” (Lat.) refers to the grey disc of the pileus.

##### Diagnosis.

*Hygrophorus griseodiscus* is distinguished from *H. brunneodiscus* by the greyish pileus with a darker grey pileus disc and larger basidiospores measuring (7)8–10(10.5) × (4)4.5–6(6.5) µm.

**Figure 7.**
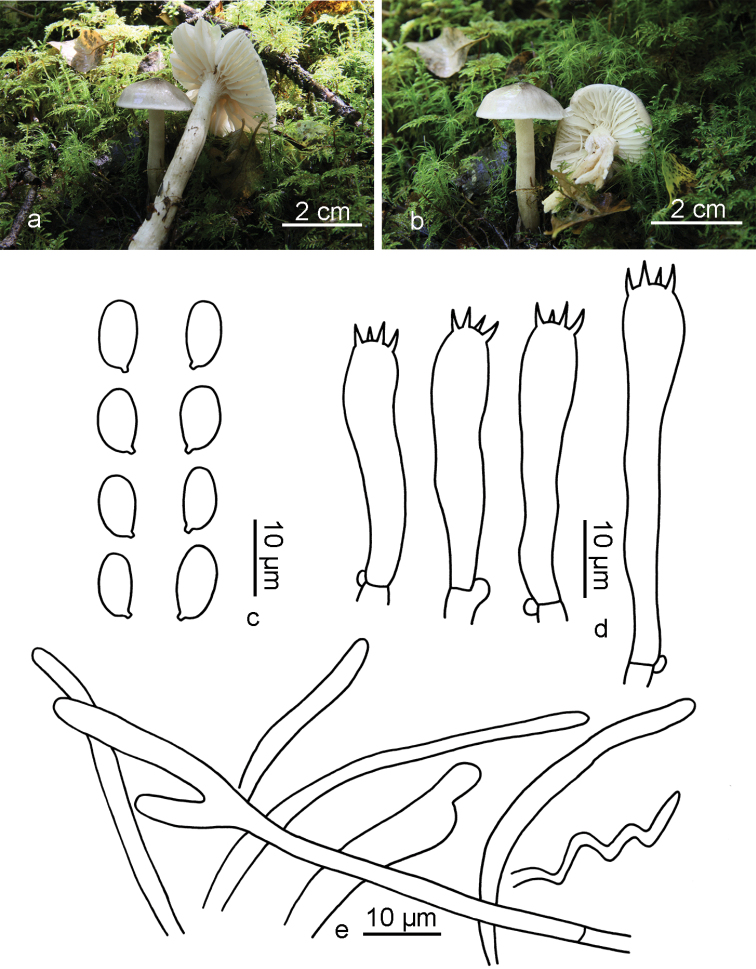
*Hygrophorus griseodiscus* (SAAS462, Holotype). **a, b** Basidiomata **c** Basidiospores **d** Basidia **e** Elements of pileipellis.

##### Description.

*Pileus* 20–45 mm broad, convex, obtusely umbonate at disc, grey to light grey (1C1, 1D1), medium to dark grey or olive grey (1E1, 1F1–4) at disc, white to pale grey (1B1) at margin, glutinous when wet; margin even, slightly involuted to extended. *Lamellae* emarginate with decurrent tooth or subdecurrent, white, thick, subcrowded, unequal, with 1–3 lamellulae between two entire lamellae. *Stipe* 40–70 × 4–6 mm, cylindrical, white to pale grey (1B1), covered with transparent glutinous materials when wet. *Context* slightly thick, white.

*Basidiospores* (7)8–10(10.5) × (4)4.5–6(6.5) µm [mean length = 9 µm, mean width = 5.2 µm], Q = 1.4–2.1, Qm = 1.74, ellipsoid, oblong to subcylindrical, smooth, hyaline. *Basidia* 29–56.5 × 7–11 µm, Q = 3.05–6(6.9), Qm = 4.39, clavate to cylindrical, thin-walled, 4-spored, with sterigmata up to 6 µm long. *Pileipellis* an ixotrichoderm, covered with a gelatinous layer; hyphae thin-walled, 2.5–6 μm wide. *Hymenophoral trama* divergent, composed of septate, thin-walled and cylindrical hyphae; hyphal cells 5.5–20 μm in width, hyaline. *Clamp connections* present.

##### Habit, habitat and distribution.

Scattered, on the ground of subalpine coniferous forest dominated by *Abies* and *Picea*, often surrounded by mosses, so far only known from Sichuan Province in Southwest China.

##### Remarks.

*Hygrophorus griseodiscus* is characterised by its convex and grey pileus with a dark grey to olive grey disc, emarginate to subdecurrent lamellae. The Asian subalpine coniferous habitat may be a helpful character for its identification.

Morphologically, *H. brunneodiscus* is distinguished from *H. griseodiscus* by the brownish pileus disc and smaller basidiospores (6.5–9.5 × 4–5 µm). *Hygrophorus cossus* differs in the greyish-white lamellae with a cream yellow tint and a thicker stipe (6–20 mm wide) (Candusso 1997, Campo 2015). *Hygrophorus discoxanthus* can be separated by the pure white pileus when young and rusty brown lamellae when mature (Candusso 1997, Campo 2015). *Hygrophorus eburneus* is different by the white pileus and the wider basidiospores (8–10 × 4.5–5.5, Qm = 1.78–1.82) (Candusso 1997). *Hygrophorus glutiniceps* is separated by the white pileus with cream or light yellow to orange tint at the disc, shorter basidiospores [(5)6–8.5(10) × (3.5)4–6 µm] and subtropical to tropical distribution. *Hygrophorus hedrychii* is distinguished by the presence of a pale orange tint on the pileus disc and an orange-pink tint on the lamellae (Larsson and Jacobsson 2004). *Hygrophorus scabrellus* is readily distinguished from *H. brunneodiscus* by its smaller basidiomata (pileus 2.4–2.8 cm broad), dark green tint on pileus and much smaller basidiospores (6.5 × 3.84 µm) (Naseer et al. 2019).

#### Hygrophorus hedrychii

Taxon classificationFungiAgaricalesHygrophoraceae

(Helen.) K. Kult, Česká Mykol. 10(4): 232 (1956)

8138DC8F-140A-5B26-820F-544333CBF0CF

Figure 8

##### Description.

*Pileus* 10–50 mm broad when dried, subglobose when young, becoming hemispherical, convex to nearly plane when mature; margin incurved when young, even, expanded to sometimes slightly upturned when mature; surface covered with a thick layer of transparent and sticky materials, white, with pinkish (7A2) to yellowish (4A2) tones or cream colour (1A2) on the disc. *Lamellae* adnate to short decurrent, white at first, changing to pinkish-white (7A2) or pale yellow (4A3) or cream colour (1A2), waxy, with 1–3 lamellulae between two entire lamellae. *Stipe* 20–85 × 3–10 mm when dried, cylindrical or nearly so, often thinner at apex, usually slightly enlarged at base, uneven, with white short fibrils at apex, sometimes longitudinally lacerated when mature, covered with a layer of transparent sticky materials, white, changing to orange white (5A2, 6A2) where touched. *Context* thick, white when young, pale to pinkish-yellow when mature, with *Cossus* smell.

*Basidiospores* (6)7–8(9) × (3.5)4–4.5(5) µm, Q = 1.5–2(2.3), Qm = 1.8, ellipsoid to subcylindrical, colourless, thin-walled, smooth. *Basidia* 30–40 × 6–9 µm, Q = 4.1–5.7, clavate, 4-spored; sterigmata 4–5 µm long; basal clamp connections common. *Pileipellis* an ixotrichoderm, composed of septate hyphae, covered with a gelatinous layer; hyphae thin-walled, 4–5 μm wide. *Stipitipellis* a trichoderm; hyphae 7–10 μm wide, light yellowish-brown intracellular pigment presented in a few hyphae when immersed in 5% KOH solution. *Hymenophoral trama* composed of septate, thin-walled and cylindrical hyaline hyphae, size 58–105 × 3–23 μm. *Clamp connections* present.

**Figure 8.**
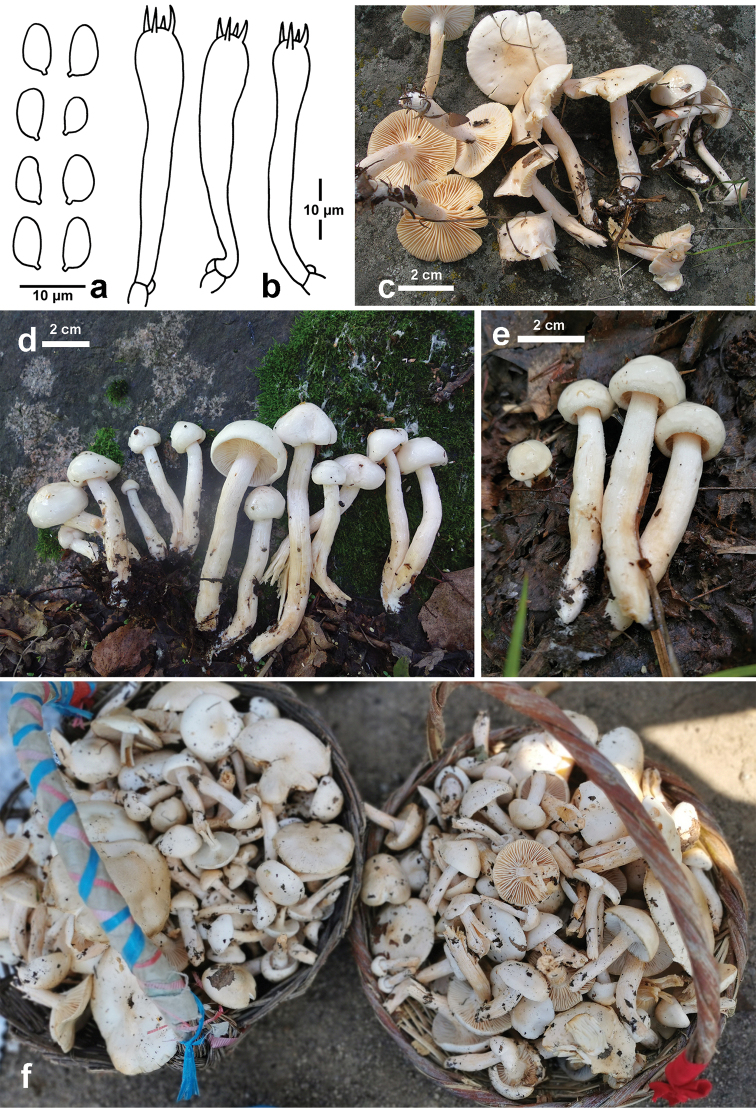
*Hygrophorus hedrychii*. **a** Basidiospores **b** Basidia **c** Basidiomata (CFSZ2851) **d** Basidiomata (CFSZ18159) **e** Basidiomata (CFSZ18269) **f** Sold at a local market, named as “baizhenmo”.

##### Habit, habitat and distribution.

Scattered to gregarious in the north temperate forests dominated by *Betula*, known from north-eastern China (this study), as well as from Europe where the species was firstly discovered.

##### Specimens examined.

China, Inner Mongolia Autonomous Region, Chifeng City, Harqin Banner, Wangyedian village, 30 August 2007, T.Z. Liu (CFSZ2851); ibidem, 8 August 2017, T.Z. Liu, Y.Q. Guang & N. Liu (CFSZ18159); Hexigten Banner, Jingpeng Town, 15 August 2017, T.Z. Liu & G.L. Yu (CFSZ18269).

##### Remarks.

Macroscopically, *Hygrophorus hedrychii* is a distinctive waxycap, which is relatively easily recognised in the field by its white pileus disc and lamellae changing to pale ochraceous pink when mature and the host association with birch. Microscopically, the Chinese specimens agree with the descriptions by Larsson and Jacobsson (2004) for the Swedish samples and Campo (2015) for the French collections. Molecular phylogenetically, they are also identical to the European species. The Chinese samples from Inner Mongolia are traded as edible fungi at some local markets. The European *H. hedrychii* was not, however, treated as an edible mushroom (Larsson, personal communication). This difference may indicate different dietary habits in different areas.

## Discussion

### Phylogeny and circumscription of subsect. Hygrophorus

In recent years, the phylogenetic framework of *Hygrophorus* has been reconstructed by Lodge et al. (2014), based on morphological characteristics and 1–4 gene regions, based phylogenetic analyses, thus, the subsect. Hygrophorus has been inclusively redefined. In order to assess the monophyly of subsect. Hygrophorus, a LSU-based phylogeny overview of the genus *Hygrophorus* and an ITS-based phylogeny overview of subsect. Hygrophorus are made in this study. According to the LSU tree, most species’ sequences of subsect. Hygrophorus are well-grouped in a clade, but three sequences with species names outside of subsect. Hygrophorus (MK278183 named as “H. cf. arbustivus”, MK278193 named as “*H. lindtneri*” and MK278194 named as “*H. marzuolus*”) are inserted in the clade of subsect. Hygrophorus. It is well-known that *H. arbustivus* and *H. lindtneri* are members of subgen. Hygrophorus/sect. Hygrophorus/subsect. Fulventes and *H. marzuolus* is a member of subgen. Camarophylli/sect. Camarophylli, according to Lodge et al. (2014). If the sequences were well-identified, it is necessary to reassess their taxonomic positions or reconsider the phylogeny framework of subsect. Hygrophorus. However, through careful checking, it was found that all three sequences were submitted by Varga et al. (2019), which mainly focused on the phylogeny reconstruction of various higher agaric taxa in a much larger scale, the species identification of the sequences not being the focus of that paper. Therefore, the correctness of the sequences’ identification is still questionable.

According to the ITS phylogenetic tree in this study, subsect. Hygrophorus is monophyletic. Thus, the concept of subsect. Hygrophorus proposed by Lodge et al. (2014) is acceptable at present, since both LSU and ITS analyses are not strongly in conflict with the circumscription. In the future, analyses, based on more samples and additional genes, are still needed.

### The species diversity of subsect. Hygrophorus

Previously, five species were confirmed as members of subsect. Hygrophorus, including *H. cossus*, *H. discoxanthus*, *H. eburneus*, *H. hedrychii* and *H. scabrellus* (Larsson and Jacobsson 2004, Lodge et al. 2014, Naseer et al. 2019). In this study, at least 13 phylogenetic species-level clades are presented as members of subsect. Hygrophorus, according to ITS-based analysis (Fig. 2), although some species are still to be verified or to be formally named.

Apart from the four new species from China, proposed in this study (*H. brunneodiscus*, *H. fuscopapillatus*, *H. glutiniceps* and *H. griseodiscus*), two species with American sequence samples (“*H. chrysaspis* Métrod” and *H. occidentalis* A.H. Sm. & Hesler) and two undescribed species, *H.* sp1 from Europe and *H.* sp2 from North America, are present as phylogenetic species under subsect. Hygrophorus. Four sequences labelled as “*H. chrysaspis*” from USA are grouped together. Although the North American samples have not been proved to be conspecific with the European *H. chrysaspis* (originally described from France) with molecular evidence due to the absence of the French sequence, the USA samples can still present a member of subsect. Hygrophorus; in addition, Hesler and Smith (1963) has also mentioned that the USA *H. chrysaspis* resembled *H. eburneus* (the type species of subsect. Hygrophorus). The American species *H. occidentalis*, originally described from Michigan (USA), is further confirmed as a member of subsect. Hygrophorus in this study (Table 2), since the isotype’s sequence (MH656479) is included; it had been regarded as a grey to fuscous member of *H. eburneus* complex, based on morphological evidence (Hesler and Smith 1963). Thus, *H. occidentalis* should be included in subsect. Hygrophours (Table 2).

It is worth mentioning that the species diversity within subsect. Hygrophorus may be underestimated for these two main reasons: 1) some species from the well-studied countries may have been left out. For example, *H. occidentalis* had been listed in the North American monograph (Hesler and Smith 1963), but it was not included in Lodge et al. (2014) since the absence of sequences at that time. 2) Many areas have been less investigated or not at all. Taking the ITS gene sequences as an example (Fig. 2), the majority sequences come from European and USA samples and very few sequences come from Africa, Oceania or South America, where there might be many taxa, different from those of Europe and North America.

**Table 2.** Morphological characteristics of known species of subsect. Hygrophorus.

Species	Pileus size (cm)	Colour of mature pileus	Colour of lamellae	Stipe width (cm)	Spore size (μm)	Host- connection	Reference
*H. brunneodiscus*	2–5	White with brownish-orange to light brown at the disc	White	0.4–0.7	6.5–9.5 × 4–5	* Quercus *	This study
*H. cossus*	3–9	Pale ochraceous grey at the centre of the pileus	Greyish-white with a cream yellow tint	0.6–2.0	7–9 × 4–5	* Quercus *	Larsson and Jacobsson 2004
*H. discoxanthus*	3–7	White with rusty-brown margin upon drying	White as young, then rusty brown	0.5–1.2	6.5–9 × 3.5–5.5	* Fagus *	Larsson and Jacobsson 2004
*H. eburneus*	2–7	White	Cream-white as young then cream-yellow	0.4–1.0	7.5–10 × 4–5.5	* Fagus *	Larsson and Jacobsson 2004
*H. fuscopapillatus*	2–3	White to pale grey at margin, pale grey to olive brown at papilla	White	0.4–0.5	(6)7–9.5(10) × (4)4.5–5.5(6)	Fagaceae	This study
*H. glutiniceps*	0.8–4	White with cream or light yellow to orange tint at disc	White when young, then changing to ochraceous or even brown	0.3–0.6	(5)6–8.5(10) × (3.5)4–6	* Castanopsis *	This study
*H. griseodiscus*	2–4.5	Grey to light grey with dark grey at disc	White	0.4–0.6	(7)8–10(10.5) × (4)4.5–6(6.5)	*Abies* or *Picea*	This study
*H. hedrychii*	3–8	White with orange-pinkish tint at centre	White with orange-pinkish tint	0.5–1.0	6.5–9 × 3.5–5	* Betula *	Larsson and Jacobsson 2004
*H. occidentalis*	2–8(10)	“hair brown” to “fuscous”, at times yellowish or smoky at disc	White then tinged cream	0.3–1(1.5)	6–8 ×3.5–5	unclear	Hesler and Smith 1963
*H. scabrellus*	2.4–2.8	Off-white with dark green	Off-white to beige	2.1–2.4	6.5 × 3.84	* Quercus *	Naseer et al. 2019

### Morphology and sequence misidentification in subsect. Hygrophorus

Some *Hygrophorus* taxa are difficult to be distinguished with the naked eye by the specific differences of macro-morphological characteristics and it will be harder when considering the infraspecific variations; the microscopic features for distinguishing *Hygrophorus* species are also limited, since they usually lack cystidia and have slight interspecific differences in the size and shape of basidiospores and basidia.

Due to the morphological similarity, misidentifications are common in subsect. Hygrophorus. For example, 15 samples with the same name as “*H. eburneus*” are included in the analysis (Fig. 2), but they are nested in different positions in the phylogram, which indicates that at least some of them are misidentified. The sequences of AY242855, AY463484, AY463485 and UDB000555 from Sweden (where the species was originally described) are identified in Larsson and Jacobsson (2004) and they should be the true *H. eburneus*. Some other European sequences, i.e. MK028422 from Switzerland, MK088116 from Hungry, MK627047 from Austria and UDB000021 labelled as “*H. discoxanthus*” from Denmark, are clustered with those real *H. eburneus* sequences from Sweden with strong support (94% BS); they should also be conspecific and treated as *H. eburneus*. These European sequences form a clade of “*H. eburneus* (Europe)”. On the other hand, there are eight sequences from USA which are gathered together in the phylogram with 95% BS, forming a sister clade to the European *H. eburneus*. Due to the close molecular similarity and the lack of adequate morphological comparison and other information, they are still treated as *H. eburneus* here as a clade of “*H. eburneus* (North America)”. However, the sequence MK737049, labelled as “H. cf. eburneus” from USA, is isolated from the true *H. eburneus*; thus, it should represent an unidentified species. In addition, the two other sequences, MK575431 from USA and DQ367904 from Canada, labelled as “*H. eburneus*” and “H. eburneus var. eburneus” respectively, are grouped as a sister clade of the Europe-Asia *H. hedrychii* clade with 100% BS. Obviously, MK575431 and DQ367904 are not the true “*H. eburneus*”, though it is not clear whether they are *H. hedrychii* or not since the species boundaries of *H. hedrychii* are not clear enough. The sequence MK564560 from USA, labelled as “*H. eburneus*”, should also be misidentified since it is clustered with the sequences (MK575247 and MF686502) of “*H. chrysaspis*”.

It is worthy to mention that, although some samples might have been misidentified, their molecular sequences can still provide some useful information for analysing the relationships amongst the samples or even potentially representing some undescribed taxa and reflecting richer species diversity.

### The distribution of Chinese species of subsect. Hygrophorus

Ecologically, according to the authors’ investigation since 2010, *Hygrophorus* species are relatively rare in the subtropical to tropical areas of Guangdong and Hainan Provinces in South China. The reports of the Chinese *Hygrophorus* are mainly concentrated in the temperate regions and in the high-elevation areas of subtropical zone (Zeng and Yang 1991, Yuan and Sun 2007, Chen and Li 2013). With the description of *H. glutiniceps*, it is scientifically confirmed from the genetic level that the distribution of *Hygrophorus* species can extend to the southernmost province of China (tropical Hainan Province).

With the application of integrative taxonomy, considering the morphology characteristics, molecular data, the symbiotic association of plants etc., it is now easier to distinguish species within subsect. Hygrophorus. Even the study of genus *Hygrophorus* has entered an era in which a mass of new species have been discovered (Jacobsson and Larsson 2007; Yu et al. 2007; Larsson et al. 2014, 2018; Endo et al. 2018; Moreau et al. 2018; Huang et al. 2018; Naseer et al. 2019). Due to the support of the Chinese government, many mushroom investigations are being carried out. It is foreseeable that, in the next few years, a large number of new species will be reported from China and the distribution of species under subsect. Hygrophorus will be expanded.

## Supplementary Material

XML Treatment for Hygrophorus brunneodiscus

XML Treatment for Hygrophorus fuscopapillatus

XML Treatment for Hygrophorus glutiniceps

XML Treatment for Hygrophorus griseodiscus

XML Treatment for Hygrophorus hedrychii

## References

[B1] Benson DA Cavanaugh M Clark K Karsch-Mizrachi I Ostell J Pruitt KD Sayers EW (2017) GenBank. Nucleic Acids Research 46(D1): D41–D47. 10.1093/nar/gkx1094 PMC5753231 29140468

[B2] Candusso M (1997) *Hygrophorus* s.l., Fungi Europaei 6. Alassio, 1–748.

[B3] Campo E (2015) *Hygrophorus*, *Hygrocybe* e *Cuphophyllus* del Friuli Venezia Giulia. Gruppo Mycologico Sacilese, 1–181.

[B4] Chen JL Li Y (2013) The checklist of species in Hygrophoraceae from China and their distribution. Journal of Fungal Research 11: 3–13, 37. [in Chinese]

[B5] Endo N Tokoo R Fukuda M Yamada A (2018) *Hygrophorus yukishiro* sp. nov., a new vernal edible mushroom from Nagano Prefecture, Japan. Mycoscience 59: 449–454. 10.1016/j.myc.2018.03.002

[B6] Gardes M Bruns TD (1993) ITS primers with enhanced specificity for basidiomycetes-application to the identification of mycorrhizae and rusts. Molecular Ecology 2: 113–118. 10.1111/j.1365-294X.1993.tb00005.x 8180733

[B7] Huang HY Yang SD Zeng NK Zhang GL Hu Y Tang LP (2018) *Hygrophorus parvirussula* sp. nov., a new edible mushroom from southwestern China. Phytotaxa 373: 139–146. 10.11646/phytotaxa.373.2.4

[B8] Jacobsson S Larsson E (2007) *Hygrophorus penarioides*, a new species identified using morphology and its sequence data. Mycotaxon 99: 337–343.

[B9] Katoh K Rozewicki J Yamada KD (2017) MAFFT online service: multiple sequence alignment, interactive sequence choice and visualization. Briefings in Bioinformatics, 1–7. 10.1093/bib/bbx108 PMC6781576 28968734

[B10] Kornerup A Wanscher JH (1978) Methuen Handbook of Colour. Eyre Methuen, London, 1–252.

[B11] Larsson E Jacobsson S (2004) Controversy over *Hygrophorus cossus* settled using ITS sequence data from 200 year-old type material. Mycological Research 108: 781–786. 10.1017/S0953756204000310 15446711

[B12] Larsson E Campo E Carbone M (2014) *Hygrophorus exiguus*, a new species in subgenus Colorati section Olivaceoumbrini, subsection Tephroleuci. Karstenia 54: 41–48. 10.29203/ka.2014.462

[B13] Larsson E Kleine J Jacobsson S Krikorev M (2018) Diversity within the *Hygrophorus agathosmus* group (Basidiomycota, Agaricales) in Northern Europe. Mycological Progress 17(2): 1293–1304. 10.1007/s11557-018-1445-y

[B14] Letunic I Bork P (2016) Interactive tree of life (iTOL) v3: an online tool for the display and annotation of phylogenetic and other trees. Nucleic Acids Research 44(W1): W242–W245. 10.1093/nar/gkw290 PMC4987883 27095192

[B15] Liu Y Tang H Li XW Chen ZH Lei ZY Xiang L (2016) Identification of medicinal and edible fungi (Agaricales) through internal transcribed spacer barcode. World Chinese Medicine 11: 791–795. [in Chinese]

[B16] Lodge DJ Padamsee M Matheny PB Aime MC Cantrell SA Boertmann D Kovalenko A Vizzini A Dentinger BTM Kirk PM Ainsworth AM Moncalvo JM Vilgalys R Larsson E Lücking R Griffith GW Smith ME Norvell LL Desjardin DE Redhead SA Ovrebo CL Lickey EB Ercole E Hughes KW Courtecuisse R Young A Binder M Minnis AM Lindner DL Ortiz-Santana B Haight J Læssøe T Baroni TJ Geml J Hattori T (2014) Molecular phylogeny, morphology, pigment chemistry and ecology in Hygrophoraceae (Agaricales). Fungal Diversity 64: 1–99. 10.1007/s13225-013-0259-0

[B17] Moreau PA Bellanger JM Lebeuf R Athanassiou Z Athanasiades A Lambert H Schwarz C Larsson E Loizides M (2018) Hidden diversity uncovered in *Hygrophorus*, sect. Aurei, (Hygrophoraceae), including the Mediterranean *H. meridionalis* and the North American *H. boyeri*, spp. nov. Fungal Biology 122: 817–836. 10.1016/j.funbio.2018.04.009 30007432

[B18] Morgan AP (1883) The mycologic flora of the Miami valley, Ohio. Journal of the Cincinnati Society of Natural History 6: 173–199. 10.5962/bhl.title.4044

[B19] Naseer A Khalid AN Healy R Smith ME (2019) Two new species of *Hygrophorus* from temperate Himalayan Oak forests of Pakistan. MycoKeys 56: 33–47. 10.3897/mycokeys.56.30280 31341398 PMC6637032

[B20] Nilsson RH Larsson KH Taylor AFS Bengtsson-Palme J Jeppesen TS Schigel D Kennedy P Picard K Glöckner FO Tedersoo L Saar I Kõljalg U Abarenkov K (2019) The UNITE database for molecular identification of fungi: handling dark taxa and parallel taxonomic classifications. Nucleic Acids Research 47: D259–264. 10.1093/nar/gky1022 PMC6324048 30371820

[B21] Peck CH (1905) Report of the state botanist 1904. Bulletin of the New York State Museum 94: 5–58.

[B22] Razaq A Ilyas S Khalid AN (2014) Taxonomic and phylogenetic affinities of *Hygrophorus chrysodon* from western Himalayan forests. Austrian Journal of Mycology 23: 21–29.

[B23] Singer R (1986) The Agaricales in modern taxonomy, 4^th^ edn. Koeltz Scientific Books, Koenigstein, 981 pp.

[B24] Trifinopoulos J Nguyen LT von Haeseler A Minh BQ (2016) W-IQ-TREE: a fast online phylogenetic tool for maximum likelihood analysis. Nucleic Acids Research 44(W1): W232–W235. 10.1093/nar/gkw256 PMC4987875 27084950

[B25] Varga T Krizsán K Földi C Dima B Sánchez-García M Sánchez-Ramírez S Szöllősi GJ Szarkándi JG Papp V Albert L Andreopoulos W Angelini C Antonín V Barry KW Bougher NL Buchanan P Buyck B Bense V Catcheside P Chovatia M Cooper J Dämon W Desjardin D Finy P Geml J Haridas S Hughes K Justo A Karasiński D Kautmanova I Kiss B Kocsubé S Kotiranta H LaButti KM Lechner BE Liimatainen K Lipzen A Lukács Z Mihaltcheva S Morgado LN Niskanen T Noordeloos ME Ohm RA Ortiz-Santana B Ovrebo C Rácz N Riley R Savchenko A Shiryaev A Soop K Spirin V Szebenyi C Tomšovský M Tulloss RE Uehling J Grigoriev IV Vágvölgyi C Papp T Martin FM Miettinen O Hibbett DS Nagy LG. (2019) Megaphylogeny resolves global patterns of mushroom evolution. Nature Ecology & Evolution 3(4): 668–678. 10.1038/s41559-019-0834-1 30886374 PMC6443077

[B26] Vellinga EC (1988) Glossary. In: Bas C Kuyper ThW Noordeloos ME Vellinga EC (Eds) Flora Agaricina Neerlandica, vol.1. A.A. Balkema, Rotterdam, 54–64.

[B27] White TJ Bruns T Lee SS Taylor J (1990) Amplification and direct sequencing of fungal ribosomal RNA genes for phylogenetics. In: Innis MA Gelfand DH Sninsky JJ White TJ (Eds) PCR protocols: a guide to methods and applications.Academic Press, San Diego, 315–322. 10.1016/B978-0-12-372180-8.50042-1

[B28] Yu FQ Xu GB Liu PG (2007) A new and noteworthy species of *Hygrophorus* from Yunnan, China. Mycotaxon 100: 169–175.

[B29] Young AM (2005) Fungi of Australia: Hygrophoraceae. ABRS, Canberra; CSIRO Publishing, Melbourne.

[B30] Yuan MS Sun PQ (2007) Pictorial Book of Mushrooms of China. Sichuan Science and Technology Press, Chengdu, 1–552. [in Chinese]

[B31] Zeng XL Yang WS (1991) Hygrophoraceae of Jilin province. Journal of Northeast Normal University 2: 71–78. [in Chinese]

